# The Nonstructural Proteins of Nipah Virus Play a Key Role in Pathogenicity in Experimentally Infected Animals

**DOI:** 10.1371/journal.pone.0012709

**Published:** 2010-09-15

**Authors:** Misako Yoneda, Vanessa Guillaume, Hiroki Sato, Kentaro Fujita, Marie-Claude Georges-Courbot, Fusako Ikeda, Mio Omi, Yuri Muto-Terao, T. Fabian Wild, Chieko Kai

**Affiliations:** 1 Laboratory Animal Research Center, Institute of Medical Science, The University of Tokyo, Tokyo, Japan; 2 International Research Center for Infectious Diseases, Institute of Medical Science, The University of Tokyo, Tokyo, Japan; 3 Institut National de la Sante et de la Recherche médicale, U758, Lyon, France; 4 Laboratory P4 INSERM Jean Mérieux, Lyon, France; Karolinska Institutet, Sweden

## Abstract

Nipah virus (NiV) P gene encodes P protein and three accessory proteins (V, C and W). It has been reported that all four P gene products have IFN antagonist activity when the proteins were transiently expressed. However, the role of those accessory proteins in natural infection with NiV remains unknown. We generated recombinant NiVs lacking V, C or W protein, rNiV(V−), rNiV(C−), and rNiV(W−), respectively, to analyze the functions of these proteins in infected cells and the implications in *in vivo* pathogenicity. All the recombinants grew well in cell culture, although the maximum titers of rNiV(V−) and rNiV(C−) were lower than the other recombinants. The rNiV(V−), rNiV(C−) and rNiV(W−) suppressed the IFN response as well as the parental rNiV, thereby indicating that the lack of each accessory protein does not significantly affect the inhibition of IFN signaling in infected cells. In experimentally infected golden hamsters, rNiV(V−) and rNiV(C−) but not the rNiV(W−) virus showed a significant reduction in virulence. These results suggest that V and C proteins play key roles in NiV pathogenicity, and the roles are independent of their IFN-antagonist activity. This is the first report that identifies the molecular determinants of NiV in pathogenicity *in vivo*.

## Introduction

Nipah virus (NiV) was first isolated in 1999 and was identified as the etiological agent responsible for an outbreak of fatal viral encephalitis in Malaysia and Singapore. During the first NiV outbreak, the virus infected both pigs and humans, in addition to a small number of cats, dogs and horses [1], [2]. NiV causes severe encephalitis with high fatality rates in humans and the virus has been responsible for highly infectious respiratory disease with low mortality in pigs. NiV has continued to re-emerge in Bangladesh and India and person-to-person transmission appeared to be the source of the outbreak in Bangladesh [3], [4], [5], [6]. Mortality rates recorded in outbreaks in Bangladesh reached 75%, which were significantly higher than that observed in Malaysia [7]. However, the molecular determinants of NiV in regard to severity of pathogenicity and ability to cross species barriers are not yet known, and thus remain important and urgent issues that need to be elucidated for understanding the disease and for development of effective vaccines. To address these issues, we have developed reverse genetics for NiV and a NiV recombinant expressing GFP [8]. Our study indicated that the receptor was important but was not the sole determinant of virus permissibility in cells.

NiV, a member of the family *Paramyxoviridae*, possesses a negative-sense, non-segmented RNA genome that is 18246 nt (Malaysian isolate) or 18252 nt (Bangladesh isolate) in length [9]. It has six transcription units that encode six structural proteins, the nucleocapsid (N), phosphoprotein (P), matrix protein (M), fusion protein (F), glycoprotein (G) and polymerase (L). Similar to other paramyxoviruses, the P gene of NiV expresses four proteins, namely P, V, W and C [10], [11]. V and W proteins are translated from the edited mRNA in which one or two non-templated G residues are inserted to the editing site during viral transcription by the polymerase protein [10]. C protein is encoded by an alternate open reading frame in the 5′ end of the P gene, and it does not have any amino acid identity with the P protein.

Viruses are subjected to various antiviral host responses upon infection, and among them, interferon (IFN) responses play important roles in early innate immunity and in the modulation of subsequent acquired immunity [12], [13]. Type I IFNs mediate their biological functions by binding to the receptor on the target cells, resulting in autophosphorylation and activation of the Janus kinases (JAKs), JAK1 and tyrosine kinase 2, leading to phosphorylation of the signaling molecules STAT1 and STAT2 [14], [15]. The phosphorylated STATs are associated with interferon regulatory factor 9 (IRF-9), and form an interferon-stimulated gene factor 3 (ISGF3) [16], [17], which activates the transcription of a large number of genes involved in the establishment of virus-resistance in the cell [18]. The V and/or C proteins of various paramyxoviruses have been shown to have IFN-antagonist activity. In NiV, all four of the P gene products have been demonstrated to have IFN antagonist activity [19], [20], [21]. The V and W proteins act on the IFN signaling by interacting with STAT1 and STAT2 [20], [21], [22], and the W protein also inhibits TLR3 pathway [23]. It has been suggested that the C protein has partial IFN antagonist activity, although the target is unclear. In many cases, viral IFN antagonist proteins have been identified as the important virulence factors [17], [24], [25], [26], [27], [28], [29]. Therefore, studies on the function of such accessory proteins of NiV should provide important insights into the pathogenesis and invaluable information for vaccine design.

We investigated the role of the accessory proteins in the NiV pathogenicity *in vivo*, using recombinant viruses lacking the accessory proteins which were constructed by reverse genetics. The results revealed that V and C proteins play key roles in NiV pathogenicity *in vivo*.

## Results

### Effect of Nipah accessory proteins on IFN signaling

**Figure 1:**
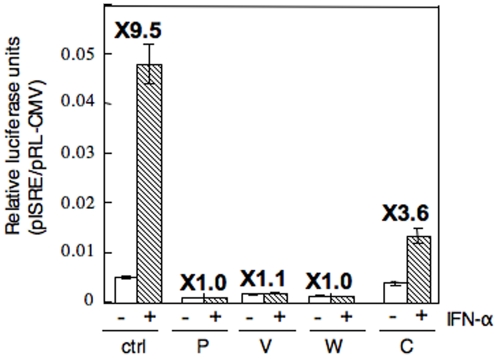
Nipah P, V, W and C proteins inhibit IFN-signaling. 293 cells were transfected with pISRE-luc, an internal control vector pRL-CMV and the indicated expression constructs (P, V, W or C) or empty vector (ctrl). At 36 h post transfection, the medium was changed to one containing 1 000 IU/ml of IFN-α. Luciferase assay was performed after 8 h incubation. All data were normalized by the expression level of *Renilla* luciferase, and the relative luciferase activity of the IFN-α treated (right shaded column) and untreated (left open column) cells is shown. The numbers at the top of the columns indicate the fold increase of luciferase activity upon IFN-α treatment. Error bars indicate the means ± the SD of three experiments.

The abilities of the V, W and C proteins of NiV to inhibit IFN signaling were analyzed by measuring the expression of the IFN-responsive reporter gene (Fig. 1). In control cells transfected with an empty vector, addition of 1 000 IU IFN-α resulted in the 9.5-fold increase of luciferase activity under the control of IFN-stimulated response element (ISRE). Transfection of expression plasmids possessing the P, V or W ORF blocked the expression of the ISRE reporter gene almost completely in response to IFN. Luciferase activity was also inhibited when the C protein expression plasmid was transfected, although the inhibition level was lower than that observed with the other constructs. These results were consistent with those reported previously [19], [20], [22].

### Generation of recombinant viruses lacking V, W or C protein

**Figure 2:**
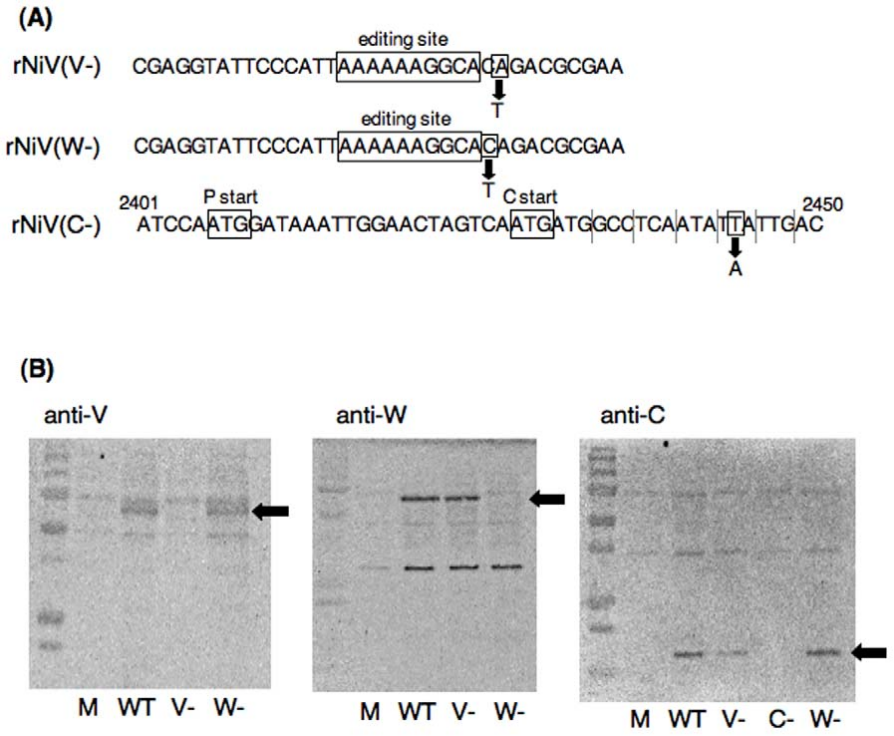
Generation of recombinant NiVs. (A) A single nucleotide change introduced into P gene ORF to produce recombinant NiVs lacking V, W or C protein. (B) Immunoblot analysis of the V, W and C proteins expressed in Vero cells infected with the parental rNiV, rNiV(V−), rNiV(W−) and rNiV(C−) (WT, V−, W−, and C−, respectively). Uninfected Vero cells (mock:M) were used as controls. Arrows indicate V (left panel), W (middle panel) or C (right panel) protein.

To examine the role of the NiV accessory proteins, we constructed the recombinant infectious clones, namely pNiV(V−), pNiV(W−) and pNiV(C−), that do not express mRNA of each accessory protein, using our previously constructed full-length NiV cDNA [8]. We first constructed altered P genes that do not express V, W or C protein using a subgenomic fragment which encompasses nucleotides 1712∼4543 (the numbers refer to the positive-sense antigenome). To prevent the expression of full-length V or W protein, a terminator codon was introduced immediately downstream of each RNA editing site by single nucleotide substitution. To prevent the expression of C protein, we introduced a terminator codon just downstream of its initiation codon by single nucleotide substitution (Fig. 2A). These substitutions were silent for the other proteins. The altered subgenomic fragment was inserted into the full-length NiV cDNA construct via unique FseI and PmeI restriction sites. All of the recombinant viruses were successfully rescued using the reverse genetics, and designated as rNiV(V−), rNiV(W−) and rNiV(C−). The substitution of the P gene in the rescued viruses was confirmed by sequencing the reverse transcriptase-polymerase chain reaction (RT-PCR) products.

To confirm that the V, W or C proteins was silenced properly in the recombinant viruses, Vero cells were infected with the parental rNiV, rNiV(V−), rNiV(W−) or rNiV(C−), and the total cell lysates extracted from the cells were subjected to Western blotting using rabbit anti-V, W and C antibodies. As shown in Fig. 2B, full-length V, W or C proteins were not observed in the cells infected with the rNiV(V−), rNiV(W−) or rNiV(C−), respectively, although the other untargeted accessory proteins were properly expressed at the same extent as those in the cells infected with the parental rNiV.

### *In vitro* growth characteristics of recombinant viruses

**Figure 3:**
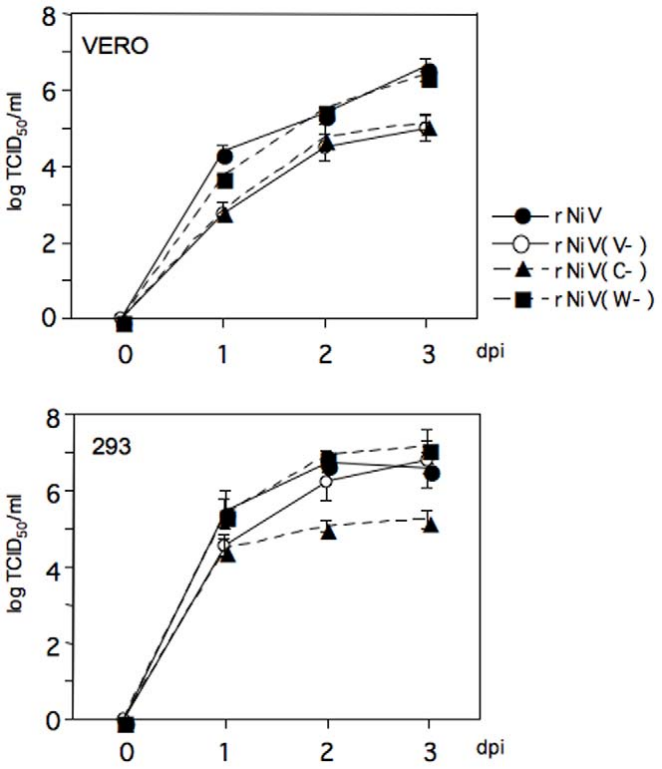
Comparison of the replication of the parent wild type (rNiV) and of the recombinant viruses, rNiV(V−), rNiV(W−) and rNiV(C−). Vero and 293 cells were infected with each virus at a moi of 0.01 pfu/cell for 1 h. Cell supernatants were collected at the indicated time points for the determination of virus titer. Error bars indicate means ± SD from three experiments.

The *in vitro* growth characteristics of the recombinant viruses in Vero and 293 cells were compared to those of the parental rNiV (Fig. 3). All recombinants grew well (≥10^5^TCID_50_/ml) in cell culture. However, in Vero cells, the single-step growth curve of rNiV(V−) and rNiV(C−) indicated that the viruses had lower growth rates and maximum titers than rNiV and rNiV(W−). In 293 cells, rNiV(V−) had a similar growth rate with the parental rNiV, although rNiV(C−) grew to lower titers.

### Inhibition of IFN-α-mediated activation of an IFN responsive promoter

**Figure 4:**
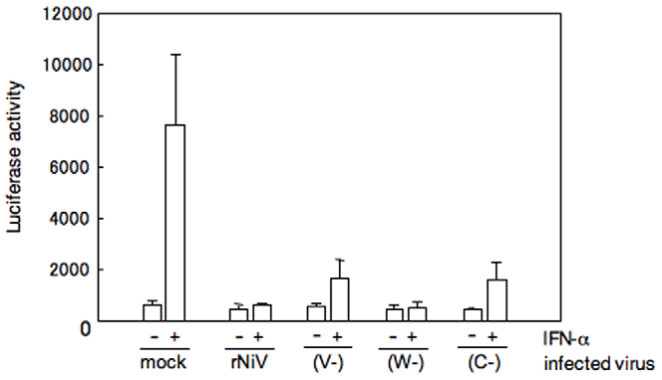
NiV inhibits the induction of luciferase expression from IFN responsive promoter. Vero cells were transfected with luciferase reporter plasmids under the control of the IFN-α promoter (pISRE-luc) and after 48 h, the cells were infected with rNiV, rNiV(V−), rNiV(W−) or rNiV(C−). At 24 h post infection, cells were treated with 1000 U of IFN-α for 24 h, lysed, and the luciferase activities were measured. Error bars indicate means ± SD of the three experiments.

The V and W proteins of NiV have been reported to inhibit the IFN signaling pathway, and C protein also has a weak inhibitory effect on IFN signaling [19], [20], [30]. We confirmed the inhibition effects on IFN signaling of the transfected proteins in our assay system (Fig. 1). As our generated recombinant viruses lacked each protein, rNiV(V−), rNiV(W−) and rNiV(C−), the viruses allowed us to examine whether the accessory proteins really functioned as antagonists of IFN signaling in infected cells. Reporter gene assays have shown that the parental rNiV completely blocked ISRE reporter gene expression, which was induced by IFN-α in infected Vero cells (Fig. 4, +/rNiV). rNiV(W−) induced suppression of the luciferase activity as strongly as the parental rNiV (+/W−). rNiV(V−) also showed strong suppressive effect on the luciferase activity. The luciferase activity in the cells infected with rNiV(C−) was also inhibited in the absence of C protein. The result indicated that NiV infection caused the suppression of IFN signaling, and that the depletion of any of the accessory proteins in the infected cells did not have influence on the suppressive effects. P protein is essential for viral gene transcription, translation and viral replication, and was abundantly expressed in cells infected with each recombinant virus. As shown in Fig. 1, P protein showed a strong suppressive effect on IFN signaling in the protein-expressed cells. Thus, P protein is sufficient for the suppressive effect on IFN response in the NiV infected cells. Alternatively, it has been previously reported that the common amino-terminal domain of NiV P, V and W was able to block IFN signaling pathways in cells expressed the domain transiently. Because both rNiV(V−) and rNiV(W−) used in this study maintained the common region upstream of the editing site, it was considered that the N-terminal part of P/V/W proteins, along with P protein, were sufficient to show a strong inhibitory effect on the IFN signaling pathway in NiV-infected cells.

### Pathogenicity of the recombinant viruses

**Figure 5:**
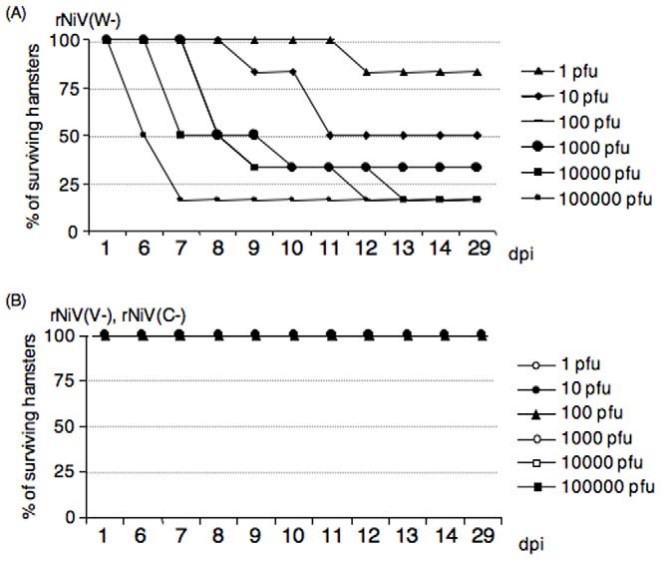
Survival curves of the hamsters infected with different quantities of the recombinant viruses. Hamsters (6 groups) were inoculated intraperitoneally with 10-fold serial dilutions of either NiV or rNiVs and survival rate of each group was observed for 30 days. (A) shows survival curves of hamsters infected with rNiV(W−) and (B) shows that of hamsters infected with rNiV(V−) and rNiV(C−).

Golden hamster (*Mesocritius auratus*) is considered to be suitable small animal model of NiV infection [8], [31]: intra-peritoneal inoculation of golden hamsters with NiV results in the development of fatal nervous signs, We compared the *in vivo* virulence of the three recombinants in the golden hamster model. Eight-week-old animals (6 per group) were inoculated with rNiV(V−), rNiV(W−) or rNiV(C−) in 10-fold dilutions (Fig. 5). rNiV(W−) induced neurological symptoms and death at similar rate to the hamsters inoculated with the parental rNiV (data in ref. 48). A total of 80% of the hamsters were killed by the rNiV(W−) infection with more than 10 000 pfu. A 50% lethal dose for rNiV(W−) observed until 12 dpi was 3.8×10^1^ pfu, which was almost the same as that for parental wt NiV (2.2×10^1^ pfu) [8]. Thus, rNiV(W−) was considered as virulent as rNiV. To confirm that the mutation made for depleting W protein expression was not reverted, we isolated viral genome from tissues of the infected animals, and sequenced them. The introduced mutation was retained, and thus rNiV(W−) was not reverted in the infected animals. In contrast to the result obtained for rNiV(W−), depletion of V or C protein dramatically reduced virulence. rNiV(V−) and rNiV(C−) induced no clinical symptoms in hamsters, and all hamsters survived, even with inoculations up to 100 000 pfu of each recombinant virus. The result clearly showed that V and C proteins play key roles in the virulence of Nipah virus while W protein does not. We analyzed an antibody response to NiV in sera from hamsters inoculated with greater than 100 pfu of the each recombinant virus at 30 dpi by ELISA, and observed the increased response (more than 1∶2000 dilutions) in all the infected animals (data not shown). Furthermore, we performed another infection experiment with 10^3^TCID_50_ of those three viruses to examine whether the viruses grew in tissues of the infected animals. The samples were taken from lung, spleen, kidney, liver, brain and heart at 6 dpi and the viral genome in the organs was detected by RT-PCR and quantified by real-time PCR. As shown in Table 1, NiV genome was detected in all collected organs of the hamsters inoculated with rNiV or rNiV(W−). The amounts of NiV genome in organs were lower in the hamsters inoculated with rNiV(W−) than in those inoculated with parental rNiV at 6 dpi. Thus, it was suggested that the rNiV(W−) grew a little slower than rNiV, and would reach to the peak after 7 dpi. In the hamsters inoculated with rNiV(V−), virus genome was not detected in any organs tested. However, the antibody response was observed in all the infected hamsters, proving that rNiV(V−) have replicated elsewhere even though at very low level. In the hamsters inoculated with rNiV(C−), the viral genome was detected in lung, spleen, kidney and liver though the numerical value was smaller than those of rNiV(W−). These data suggested that rNiV(V−) and rNiV(C−) replicated in the hamsters, but the growth of the viruses remained below the threshold levels to induce clinical symptoms in hamsters. In addition, lung appeared to be a site of extensive virus replication.

**Table 1:** Quantification of NiV N genome in hamster tissues.

	ID	lung	spleen	kidney	liver	brain	heart
mock	1	0.002	0.001	0.033	0.003	0.000	0.000
	2	0.003	0.000	0.002	0.003	0.000	0.001
rNiV	3	**1562.082**	**3.576**	**124.812**	**7.491**	**28.318**	**8.921**
	4	**2237.473**	**12.843**	**79.734**	**35.968**	**71.092**	**65.189**
rNiV(V−)	5	0.001	0.003	0.003	0.004	0.001	0.001
	6	0.001	0.000	0.001	0.002	0.004	0.001
rNiV(C−)	7	**0.937**	**0.251**	**0.112**	**0.010**	0.000	**0.012**
	8	**0.906**	**0.094**	**0.032**	**0.036**	0.001	0.003
rNiV(W−)	9	**186.666**	**8.124**	**41.107**	**2.785**	**2.531**	**8.732**
	10	**32.053**	**0.361**	**5.521**	**0.281**	**0.078**	**0.129**

Bold letters: NiV N detected (rounded down to second decimal place).

**Figure 6:**
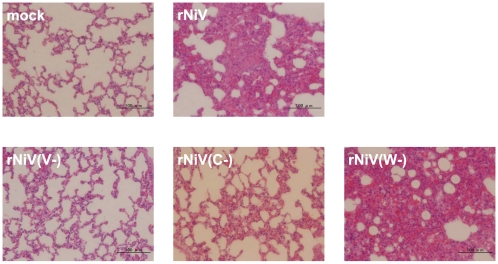
Histopathology in lungs. The thin sections of kidney samples obtained from hamsters infected with rNiV, rNiV(V−), rNiV(C−) and rNiV(W−), were stained with hematoxylin and eosin. Severe inflammation lesions were observed in lung from rNiV or rNiV(W−) infected hamsters. In lung of rNiV(C−) infected hamsters, mild infiltration and inflammation were observed. The lung from rNiV(V−) was normal.

Histopathologically, large and severe inflammatory lesions were observed in lung collected from rNiV(W−) infected hamsters at same extent of that from parental virus infected animals (Fig. 6). These observations were similar to the previous findings in the hamsters infected with wild type NiV [31]. In contrast, the lungs of rNiV(V−) infected hamsters were normal in every aspect. In lung of rNiV(C−) infected hamsters, infiltration of inflammatory cells were observed to a slight degree.

## Discussion

Non-structural proteins of a large number of viruses are known to have inhibitory effects on IFN signaling. In the case of NiV, V proteins have been demonstrated to subvert IFN responses by sequestering STAT1 and STAT2 in high molecular mass cytoplasmic complexes without inducing degradation and the complex formation prevents IFN-induced STAT tyrosine phosphorylation [20], [30]. The N terminal domain of the V protein, from residues 100 to 160, is responsible for the activity and the region is shared by the W and P proteins [20], [21], [30]. Furthermore, the single amino acid at residue 125 plays a critical role in IFN antagonism exerted by NiV V protein [32]. The NiV W protein has a nuclear localization signal in its unique terminal domain and sequesters STAT1 in the nucleus, thus creating both a cytoplasmic and a nuclear block for STAT1, and it inhibits the transcription of antiviral genes under control of ISRE promoters activated by ISGF3 [21], [33]. Thus, P, V and W proteins act as antagonists for IFN-responses by sequestering STAT proteins, with the W protein being the most efficient and the P protein the least [34]. NiV V and W proteins can also inhibit virus-induced activation of the IFN-β promoter and IRF3-responsive promoter (ISG54 promoter) and only the W protein shows strong inhibition of promoter activation in response to the stimulation of Toll-like receptor 3 [23]. NiV C protein was also suggested to have weak IFN antagonist activity, but the target is unclear [19].

These antagonistic activities of IFN-signaling of P, V, W and C of NiV were all demonstrated by the experiments using transiently expressed proteins following transfection with plasmid DNA, and we also confirmed strong inhibition of IFN response with P, V, W and C proteins expressed by plasmids in the reporter gene assay (Fig. 1). However, whether these proteins actually function in the infected cells was not clarified. To address this question, we generated recombinant viruses lacking accessory proteins. rNiV(V−) and rNiV(W−), as well as parental rNiV, induced strong suppression of the IFN response observed by the same reporter gene assay. Particularly, the W knockout virus rNiV(W−) showed the complete inhibitory effect. It indicated that the domain unique to W protein does not play a role on the suppressive effect in the infected cells, or that the suppressive effects of the other remained proteins are sufficient to show the complete inhibitory effects. Both recombinant viruses used in this study expressed intact P protein and the N terminus of V or W proteins containing the STAT-binding region, which is upstream of the editing site. Thus, it is understandable that our recombinant viruses that lacked full-length V or W proteins still retained IFN antagonist activities that were comparable to rNiV. This result is in agreement with the previous studies showing that the N-terminal domain of these proteins transiently expressed in cells has strong IFN-antagonist activities [35]. The C knockout virus rNiV(C−), in which the C protein is completely removed, also induced strong suppression of the IFN response in the infected cells. This is not surprising because P, V, and W proteins were not depleted from rNiV(C−). This result may concur with the previous findings that indicated that measles virus (MV) and rinderpest virus (RPV) C protein had no effect or had a weaker effect on IFN signaling in the context of viral infection [13], [36]. It seems likely that the C protein may have the potential to inhibit IFN signaling, as suggested by the results of the experiments with C protein-expressed cells, but the effect is probably not significant in the virus infected cells.

Previously, we reported that the rNiV induced severe encephalitis and death in hamsters in the dose-dependent manner similar to those induced by the parental NiV [8]. The virulence of rNiV(W−) tested in the present study was not altered as compared to those of rNiV and NiV, and the virus killed animals in a similar dose-dependent manner. However, the virulence of rNiV(V−) and rNiV(C−) were dramatically reduced and they did not kill any of the hamsters even when the animals were inoculated with as much as 10^5^ pfu of the viruses.

Viral genome was detected in some organs collected from rNiV(C−) infected hamsters, but not in those from rNiV(V−) infected hamsters. Anti-NiV antibody response was induced following infection with each of the viruses, including rNiV(V−), indicating that rNiV(V−) replicated somewhere in the animal body although the viral genome was not detected in tested organs of hamsters inoculated with low titers of rNiV(V−) at 6 dpi. Although rNiV(W−) showed severe pathogenicity and killed the infected hamsters at similar rates compared with those with the parental rNiV, virus growth in organs of the infected animals were lower, or slower, than those in the rNiV infected animals. Thus, W protein probably affects the virus growth in organs of the infected animals, even though the effect is weaker than V and C. However, since NiV pathogenicity is extremely strong, the lower amount of replicated rNiV(W−) in the body seemed to be sufficient to kill the animals. These observations clearly showed that the V and C proteins, but not the W protein, play a significant role in NiV pathogenicity.

It should be noted that although rNiV(V−) and rNiV(C−), in addition to rNiV(W−), retained the strong antagonistic activities on IFN-signaling in their infected cultured cells (Fig. 4), rNiV(V−) and rNiV(C−) lost virulence *in vivo* (Fig. 5). These results suggest that the major roles of the V and C proteins on *in vivo* pathogenicity are probably not implemented through the inhibitory activities on IFN signaling.

Regarding the growth features in cultured cells, the three recombinant viruses replicated efficiently in Vero and 293 cells, although their maximum titers differed. Among the recombinant viruses, only rNiV(W−) showed a similar growth curve to the parental rNiV and reached the same maximum titer in both Vero and 293 cells. Growth of rNiV(C−) was slower, and its maximum titer was lower than that of the parental rNiV. The less efficient growth feature of the rNiV(C−) was not related to its IFN-resistant properties because Vero cells do not produce IFN whilst 293 cells do. Previous studies indicated that the paramyxovirus C proteins are multifunctional proteins. The MV C protein regulates viral RNA synthesis [37], in addition to inhibiting the IFN response [38]. Sendai virus C proteins were reported to be relatively versatile and were considered to play a role in inhibiting viral transcription and replication [39], [40], and in virus assembly and budding [35], [41], in addition to the effect on IFN signaling [42], [43]. The growth rate of the C knockout Sendai virus was slower than that of the parental virus and the yield was lower [41]: it showed reduced pathogenicity in mice in comparison to the parental virus [44], which was similar to our observation using rNiV(C−). Thus, the C protein of NiV might also contribute to virus assembly and affect virus growth in certain cells. Further study is required to clarify these issues.

The mechanism by which the deletion of the V protein of NiV confers attenuation phenotype also remains to be elucidated. rNiV(V−) has revealed different features of viral growth in two kinds of cells. It grew and reached the same maximum titer as the parental virus in 293 cells but had slower growth and lower yield in Vero cells. This result suggests that the NiV V protein may play an unknown role in virus replication which affects Vero cells but not 293 cells. Several reports discuss the function of V proteins *in vivo* using V-knockout paramyxoviruses. It has been reported that the infection rate of T cells with V-knockout canine distemper virus (CDV) was reduced and the virus failed to deplete the T-cell fraction of peripheral blood mononuclear cells (PBMC) in ferrets (*Mustela putorius furo*) [45]. This V-knockout CDV unregulated not only IFNs but also cytokines, such as tumor necrosis factor alpha, IL-6, IL-2 and IL-4. Similarly, in MV, the V protein was reported to interfere with STAT3-dependent IL-6 signaling [46]. Therefore, V protein is likely to have pleiotropic anti-immune response functions and thus depletion of this protein may attenuate the virus *in vivo*.

The effects of IFN antagonistic activities of V and W proteins on *in vivo* pathogenicity could not be analyzed in our study because the STATs-interacting domain of V/W resides in the region shared by P protein. Since P protein is essential for viral RNA synthesis, we were unable to delete it when generating recombinant viruses. However, a recent report has revealed that Ser130 and Ser131 of the NiV P protein are responsible for binding to STAT1 and STAT2 and normal IFN signaling interference, and mutations introduced on these sites has no effect on its ability to support minigenome transcription and replication [47]. Therefore, the role of the IFN antagonistic activities of V/W proteins on *in vivo* pathogenicity can now be assessed by producing recombinant viruses which do not inhibit STAT-dependent IFN antiviral response.

In this study, we found that the depletion of the V or C protein significantly reduced the pathogenicity of NiV *in vivo*. Such a drastic decrease in pathogenicity has not been reported in any members of the paramyxoviruses. On the contrary of the previous speculation, the attenuation mechanism observed in the rNiV(V−) and rNiV(C−) are not caused by the loss of IFN antagonist activities due to these proteins, because rNiV(V−) and rNiV(C−) retained similar activities in infected cells. Further studies should clarify the unknown mechanisms and the possibility of attenuated viruses serving as effective vaccine candidates should also be elucidated.

## Materials and Methods

### Ethical treatment of animals

All animal experiments were approved by Comité régional d'ethique pour l'expérimentation animale Rhone Alpes.

### Cells

Vero (monkey kidney), CV-1 (monkey kidney) and 293 (human kidney) cells were maintained in Dulbecco's modified Eagle medium (DMEM) (GIBCO BRL) containing 5% fetal bovine serum (FBS), L-glutamine and antibiotics.

### Rescue of recombinant virus from cDNA

The plasmid pNiV(6+), containing the full-length NiV cDNA flanked by T7 RNA polymerase sequence, has been described previously [8]. To construct the full-length genome plasmid for the recombinant virus-lacking C protein, the nucleotide 2444T (the number refers to the positive-sense antigenome) was changed to ‘A’ to introduce a terminator codon. For the recombinant virus-lacking V or W protein, a terminal codon was introduced by substitution downstream of each editing site.

The V, W or C protein-lacking viruses designated ‘rNiV(V−)’, ‘rNiV(W−)’ and ‘rNiV(C−)’, were rescued from these cDNA in accordance with the procedure described previously [8]. The rescued viruses were passaged once in Vero cells and stored at −80°C.

### Western blotting

Vero cells were infected with rNiV, rNiV(V−), rNiV(W−) or rNiV(C−) at a multiplicity of infection (moi) of 0.1. The cells were collected at 24 hpi, and total cell lysates were extracted using Trizol reagent (Invitrogen) in accordance with the instructions of the manufacturer. Cellular proteins are isolated from the phenol-ethanol supernatant obtained after ethanol precipitation of DNA. The lysates were boiled in 2× sodium dodecyl sulphate (SDS)-loading buffer with 5% beta-mercaptoethanol for 5 min and separated on 14% polyacrylamide gels. Resolved proteins were transferred to polyvinylidine difluoride membranes and blocked overnight with 5% skimmed milk in (Tris-buffered saline Tween) TBST (0.05% Tween 20 in TBS). Blots were incubated with primary antibodies for 60 min at room temperature and washed six times with TBST. The primary anti-V, W and C antibodies were produced in rabbits by immunization of peptides which were designed from unique regions of those proteins. Subsequently, the blots were further incubated with the secondary horse radish peroxidase (HRP)-conjugated anti-rabbit antibody for 30 min. After washing, bands were visualized using the ECL™ Western blotting detection system (Amersham).

### Virus growth

Vero and 293 cells (1×10^6^/6-well plate) were infected with rNiV, rNiV(V−), rNiV(W−) or rNiV(C−) at an moi of 0.01 for 1 h. The inoculum was removed and the cells washed once with medium and then incubated in DMEM +2% FBS. Cells and supernatants of wells infected with these viruses were harvested 24 h, 48 h and 72 h later, centrifuged and then stored at −80°C. The TCID_50_/ml of each sample was measured using standard methods. The experiment was repeated twice.

### Infection of hamsters

Golden hamster (*Mesocricetus auratus*) appears to be a reliable model of NiV infection [8], [31]. Eight-week-old golden hamsters were injected intraperitoneally with recombinant NiV after being anaethetised with isoflurane (Aerrane; Baxter S.A.). The animals were housed in ventilated containers equipped with high efficiency particulate air (HEPA) filters in the BSL-4 laboratory.

### Real-time PCR analyses

The tissue samples, dissected from the infected hamsters, were homogenized in 500 µl of Trizol reagent and total RNAs were extracted in accordance with the instructions of the manufacturer. First strand cDNA was synthesized using toal RNA and random primer. For real time RT-PCR, total RNA was reverse-transcribed, and then it was PCR-amplified by ABI Prism 7900HT (Applied Biosystems, USA) using SYBR Premix Ex TaqII (Takara, Japan). Specific HPRT (hypoxanthine-guanine phosphoribosyltransferase) primers were used as an internal control. The data were analyzed with Sequence Detection Systems ver 1.7a software (Applied Biosystems). The expression levels of the target genes were calculated using the threshold cycle time (Ct), the first cycle number at which emitted fluorescence exceeds 10× the standard deviation (SD) of base-line emission as measured in the cycles of PCR. Standard curve was generated using known cDNA concentration (10-fold dilution from 10 ng ∼1 pg/reaction). Normalized results were expressed as the ratio pg RNA of NiV N gene to pg RNA of the HPRT gene.

### Reporter gene assays

The sequences coding for P, V, W and C proteins were amplified by PCR with primers containing SalI sites at both ends of the fragment using Thermo-Start DNA polymerase (AB gene). The PCR products were digested by SalI and cloned into pCMV-myc vector (BD bioscince). For luciferase assays, Vero cells were transfected with three plasmids, namely: pCMV-myc construct of each of the relevant genes or a pCMV-myc empty vector as a control, a plasmid containing the luciferase gene downstream to an IFN-stimulated response element (pISRE-luc) and a construct encoding the *Renilla* luciferase protein (pRL-luc) using lipofectamine LTX (Invitrogen). To assay virus-infected cells, Vero cells were transfected with pISRE-luc and pRL-luc two days prior to infection; the recombinant viruses were then infected at a moi of 2. On the following day, the medium was replaced with medium containing 1 000 IU of IFN-α, and incubation was continued for 24 h. The cells were harvested to analyze luciferase activity. Luciferase assays were performed using the *Renilla* luciferase assay system (Promega) in accordance with the instructions of the manufacturer.

### Histopathological examination

Organs removed at autopsy were fixed in 10% formalin, dehydrated and embedded in paraffin using routine techniques. Thin sections were stained using hematoxylin and eosin.
